# VEGF-A-induced changes in distal outflow tract structure and function

**DOI:** 10.1007/s00417-023-06252-5

**Published:** 2023-10-13

**Authors:** Jannis Oltmann, Mark Morell, Mohamad Dakroub, Raoul Verma-Fuehring, Jost Hillenkamp, Nils Loewen

**Affiliations:** 1 Department of Ophthalmology, University Hospital Würzburg, Josef-Schneider-Straße 11, 97080 Würzburg, Germany; 2 ARTEMIS Eye Centers of Frankfurt, Hanauer Landstr. 147, 60314 Frankfurt, Germany

**Keywords:** VEGF-A, Distal outflow tract, Outflow facility, SD-OCT, Porcine eyes

## Abstract

**Purpose:**

To investigate changes in distal outflow tract vessels caused by VEGF-A and their impact on outflow.

**Methods:**

We compared VEGF-A perfused porcine anterior segments with and without trabecular meshwork (TM) to control eyes. In the first experiment (*n*=48), we analyzed live changes of the outflow tract with spectral-domain optical coherence tomography (SD-OCT) over 3 h and reconstructed them in 3D. In a second experiment (*n*=32), we measured the intraocular pressure (IOP) variation in response to VEGF-A over 48 h and computed the outflow facility.

**Results:**

VEGF-A increased the vessel volume of the distal outflow tract by 16.8±10.6% while control eyes remained unchanged (0.5±6.8%). Volume changes occurred within the first 100 min before plateauing at 140 min. VEGF-A enhanced the outflow facility in eyes without TM by 38.6±25.5% at 24 h as compared to controls (*p*<0.05).

**Conclusion:**

VEGF-A dilated vessels of the distal outflow tract and increased the outflow facility even after TM removal, pointing to a regulatory mechanism independent of proximal structures.

**Supplementary Information:**

The online version contains supplementary material available at 10.1007/s00417-023-06252-5.


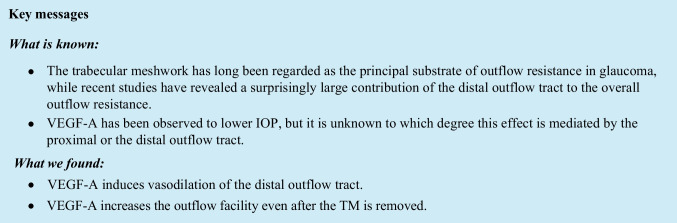


## Background

Lowering intraocular pressure (IOP) is currently the only proven strategy to delay the progression of glaucoma [1, 2], a progressive and irreversible disease of the optic nerve that often leads to blindness. Several surgical treatment modalities target the trabecular meshwork (TM) that guards the proximal end of the conventional outflow system. The TM has long been considered the primary cause of increased outflow resistance in glaucoma. However, recent studies have shown that most canal-based glaucoma surgeries fail to lower IOP to the level of episcleral venous pressure [3–6], the lowest theoretical limit [7]. Ex vivo studies have now shown that approximately 50% of the outflow resistance in the eye must be located further downstream of the trabecular meshwork and is generated by an as-yet unidentified mechanism [8–12]. In addition to these ex vivo studies, our findings in patients who underwent ab interno trabeculectomy (AIT), a minimally invasive glaucoma surgery that removes the TM, indicated an increased distal outflow resistance with a concerning rate of higher than expected IOP [13].

Recent studies suggest that VEGF-A may play a regulatory role in conventional outflow, including both the proximal and distal outflow pathway: VEGF-A is secreted by human TM cells in culture [14], and the level increases in response to mechanical stress [15]. The outflow facility decreased when VEGF-A-VEGFR-2 interaction was blocked in enucleated mouse eyes [15]. The isoform 121 of VEGF-A has been shown to increase the outflow facility by interacting with VEGFR2 [16]. In addition to these findings, clinical data show an increased risk of sustained IOP elevation in patients receiving intravitreal injections of anti-VEGF therapeutics [17].

Based on these studies, we set out to investigate the effects of VEGF-A on the morphology and function of the distal outflow pathway. We hypothesized that VEGF-A would lead to vasodilation of this structure, detectable by spectral-domain optical coherence tomography (SD-OCT), thereby increasing the outflow facility. Our findings provide new insights into the mechanisms involved in the regulation of ocular outflow facility, with potential implications for the treatment of glaucoma.

## Materials and methods

### Study design

This study was designed to determine the effects of VEGF-A on vascular morphology and function of the distal outflow pathway. All experiments were performed in porcine eyes because of some similarities between porcine and human eyes, a considerable amount of existing data on their use in ex vivo perfusion cultures, and their freshness and availability compared to human donor eyes [9, 16, 18–21]. To detect a vessel diameter change of at least 10%, we calculated the sample size based on a testing power of 80% and an alpha of 0.05. We assumed a standard deviation of 6.6% in control eyes, which was derived from our preliminary data (not shown). The minimum sample size was 5 eyes per group, but we increased it to 12 eyes per group to enhance the power.

We imaged 48 porcine eyes using an investigational deep tissue penetration SD-OCT to quantify morphological changes as described below. The eyes were divided into four groups of 12 eyes each (VEGF-A, VEGF-A_AIT_, controls, controls_AIT_). In VEGF-A_AIT_ and controls_AIT_, the TM was removed over 360 degrees by ab interno trabeculectomy (AIT) as described before [20, 22, 23]. We perfused the eyes for 3 h and obtained SD-OCT images at regular intervals.

To assess how the outflow facility was impacted, we used 32 eyes in four groups, VEGF-A, VEGF-A_AIT_, controls, controls_AIT_, each containing eight eyes. We measured IOP and compared the computed facility changes for 72 h.

### Preparation and incubation

Porcine eyes were obtained from a local abattoir and processed and mounted in perfusion culture within 2 h of slaughter, as described previously. Eyes were randomly selected from the bag and allocated to groups in a blinded fashion. Eyes with signs of pathology or trauma were excluded. Allocation to AIT was also alternated. Three investigators conducted the experiments over a period of 6 months, using fresh porcine eyes obtained on different days of the week to reduce bias from factors such as butchering technique or lot. Institutional Animal Care and Use Committee approval was not required because these animals were not sacrificed for research. Briefly, the extraocular tissues were removed, the eyes were disinfected in 5% povidone-iodine ophthalmic solution (Betadine 5%, Fisher Scientific, NC9771653), and hemisected along the equator under aseptic conditions. Anterior porcine segments were secured in a perfusion dish. To allow sufficient time for equilibration, all eyes were perfused for 24 h with Dulbecco’s modified Eagle’s medium supplemented with 1% fetal bovine serum (FBS, 10438026; Thermo Fisher Scientific, Waltham, MA) and 1% antibiotic and antifungal (15240-062; Thermo Fisher Scientific, Waltham, MA). Eyes intended for SD-OCT imaging were perfused with a microinfusion pump (70-3007; Harvard Apparatus, Holliston, MA, USA) at 3 μl/min, while eyes intended for outflow facility measurements were perfused at 6 μl/min, as we have done previously to mimic a glaucomatous condition. Eyes in the treatment group were supplemented with recombinant human VEGF-A (MGC70609, Prospec-Tany TechnoGene Ltd, Ness Ziona, Israel) at a concentration of 13.33 ng/mL after completion of baseline measurements.

### SD-OCT image caption and processing

A fixed pressure, gravity-based infusion was chosen, with IOP set by a water column height of 30 cm, equivalent to 22 mmHg. The IOP had to be kept as stable as possible to avoid changes due to fluctuating vascular inflation and to prevent hypotony with unstable mounts, which can occur after AIT. All eyes were allowed to equilibrate with the control perfusate for 1 h. We used SD-OCT imaging (Envisu R2210, Leica, Morrisville, NC, USA) for repeatable, noninvasive, high-resolution assessment of tissue morphology. Scan cubes were 6.0 mm along the limbus, 4.0 mm wide, and 1.6 mm deep and were acquired every 20 min for a total of 3 h. The imaging head was tilted to keep the 10-mm telecentric lens perpendicular and parallel to the limbus. All images were processed using image processing software (ImageJ 1.50b, http://imagej.nih.gov/ij, Wayne Rasband, NIH) to remove some of the background noise, extract signal voids from the stacks, and convert to TIFF files. All 10 data cubes per eye were then imported into Avizo 3D software (v2021.2, FEI; ThermoScientific) for denoising, 3D reconstruction, and realignment of the imaged structures. Areas with vessels were automatically detected, and their volumes were measured. For each eye, the volumes at all ten time points were used for linear regression. This regression was used to determine the relative volume change.

### Outflow facility measurements

IOP was measured continuously for 72 h with pressure transducers (Deltran II: DPT-200; Utah Medical Products, Midvale, UT, USA), recorded (FE224, PL3508/P, MLA1052; ADInstruments, Sydney, Australia), and analyzed (LabChart 7; ADInstruments). The IOP was monitored for 48 h after 24 h of control perfusion for each eye. This allowed the TM to resume normal function with a stable baseline IOP prior to the treatment. The total observation period was 72 h. Outflow facility was calculated for each eye at baseline, as well as 24 h and 48 h after AIT and the beginning of VEGF-A perfusion.

### Statistical analysis

Data were collected and analyzed using Excel (Microsoft, Redmond, WA, USA) and SPSS Statistics (version 27, IBM, Armonk, NY, USA), respectively. Means and standard deviations were reported for all parameters. Normal distribution was presumed based on medical data evidence and verified using the Kolmogorov-Smirnov test. The percentage of volume change over time for each eye was calculated by fitting a linear regression to all data points. This method was more reliable than comparing only the baseline and 3-h values. The volume changes between groups were then compared using ANOVA for normally distributed data and the Mann-Whitney U test for non-normally distributed data. To confirm these findings, a Repeated Measures (RM)-ANOVA was used to evaluate the volume changes over time. A potential interaction between AIT and VEGF-A was assessed using a Two-way-ANOVA. *P* values of 0.05 or less were considered statistically significant.

## Results

### VEGF-A induces dilation of distal outflow tract vessels

**Fig. 1:**
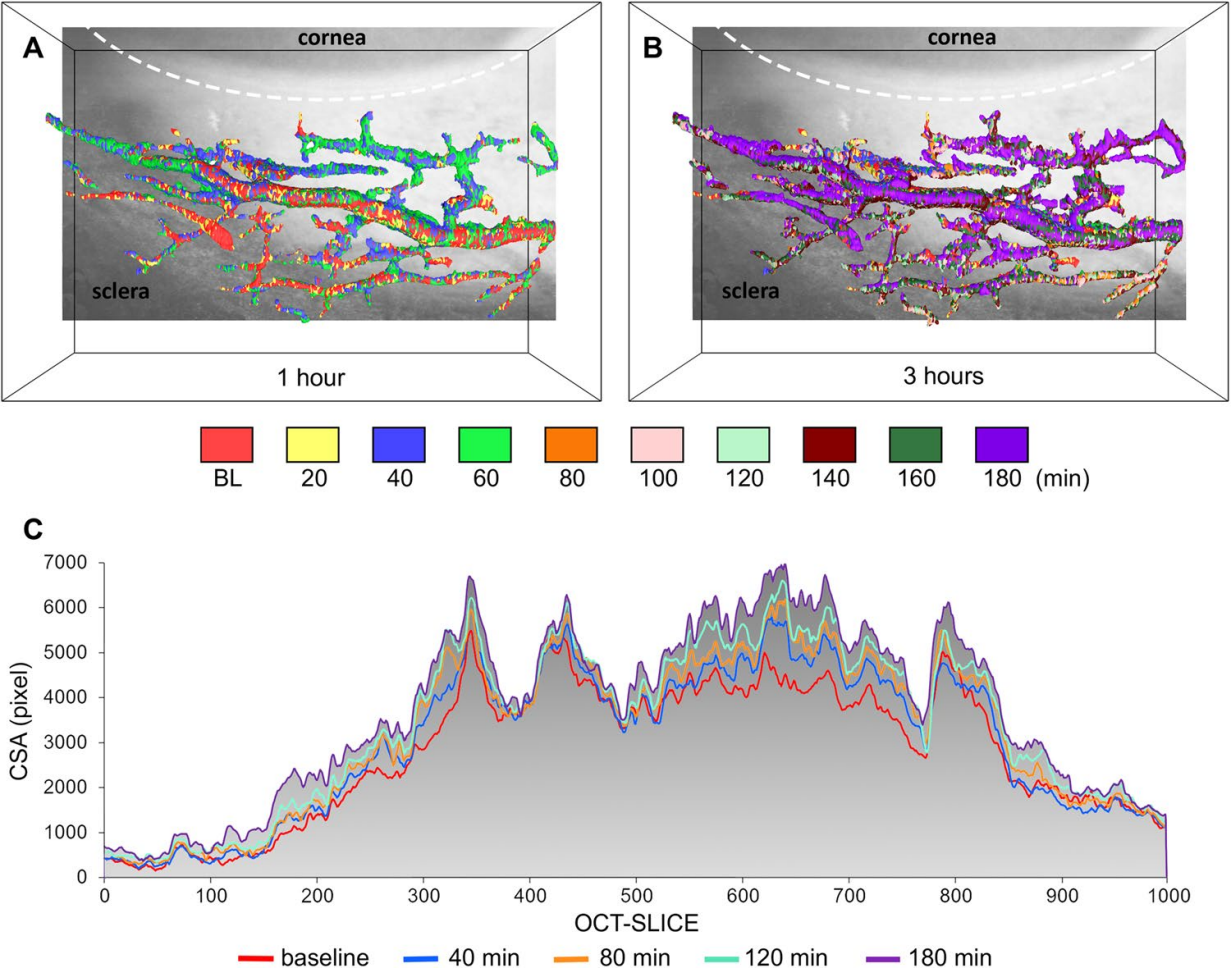
Vessel volume increases over time with VEGF-A treatment. Example of a 3D reconstructed porcine distal outflow tract with all acquired OCT images. This eye was treated with VEGF-A after AIT. Images are 6-mm parallel to the limbus, 4-mm perpendicular to the limbus, and 1.6-mm deep. Baseline vessel volume is shown in red. The additional volume for each image is the overlap of the vessels as indicated below the images. Superimposed volume at 1 h (**A**) and 3 h (**B**). Vessel volume was calculated by measuring the vessel covered area or CSA per OCT slice. Data for five time points are shown in **C** (CSA in pixels). Volume was calculated as the area under the curve of CSA and expressed in voxels. This particular eye showed a 25% increase in volume

We were able to visualize the distal outflow tract without contrast. The OCT stacks were properly aligned using Avizo 3D software. Vessel volumes and morphology were successfully calculated for all ten aligned image time points per eye. An example of the aligned scleral and episcleral vessels is shown in Fig. 1A, B and Extended Data Fig. S1. For each OCT slice, the cross-sectional area (CSA) of the vessels is shown in Fig. 1C.

**Fig. 2:**
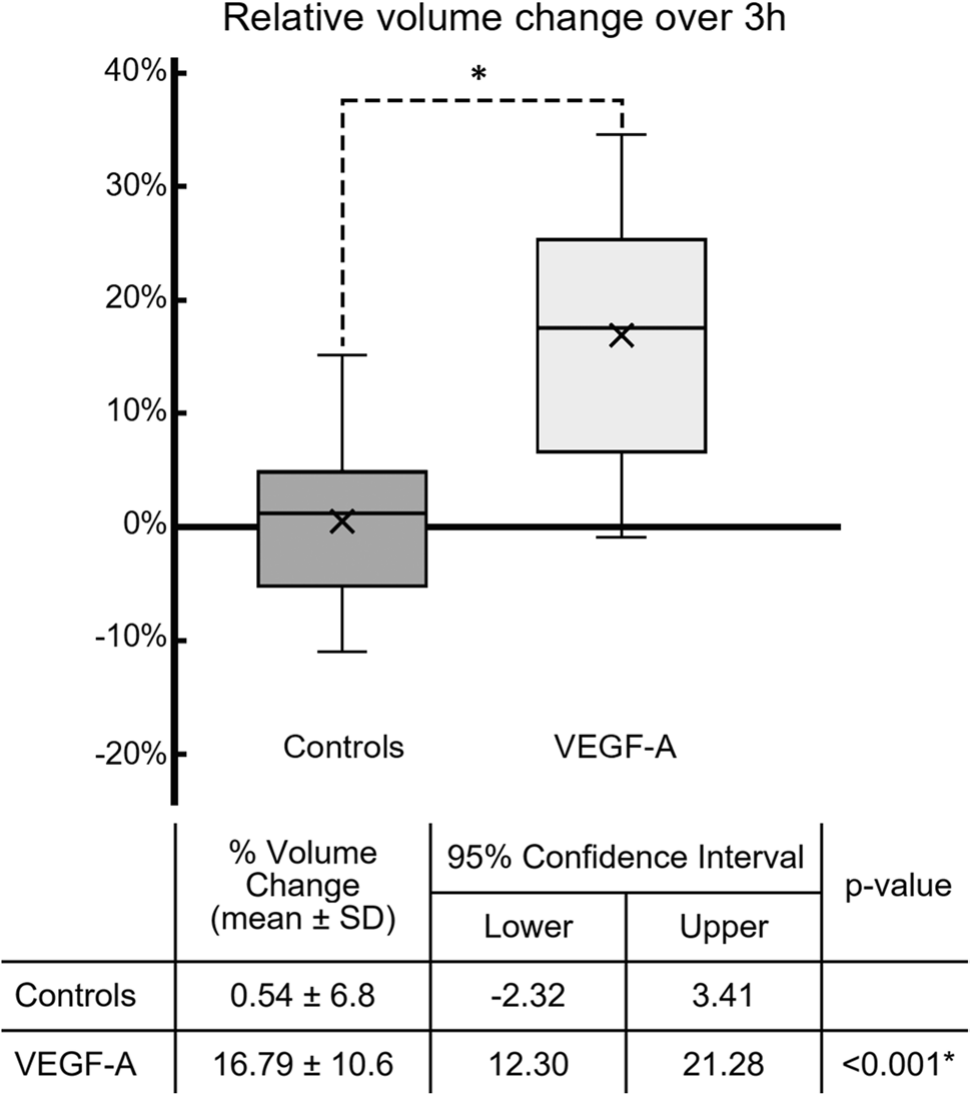
Distal outflow tract volume increases under the influence of VEGF-A. Volume changes are shown for all eyes relative to their baseline volume (*n*=48). The vessel volume increase was significantly higher in VEGF-A treated eyes (16.8% ± 10.6%) compared to controls (0.54 ± 6.8, group difference: *p*<0.001, ANOVA). Box-plot indicates the relative volume change, *x*=mean, SD= standard deviation. *p<0.001

**Fig. 3:**
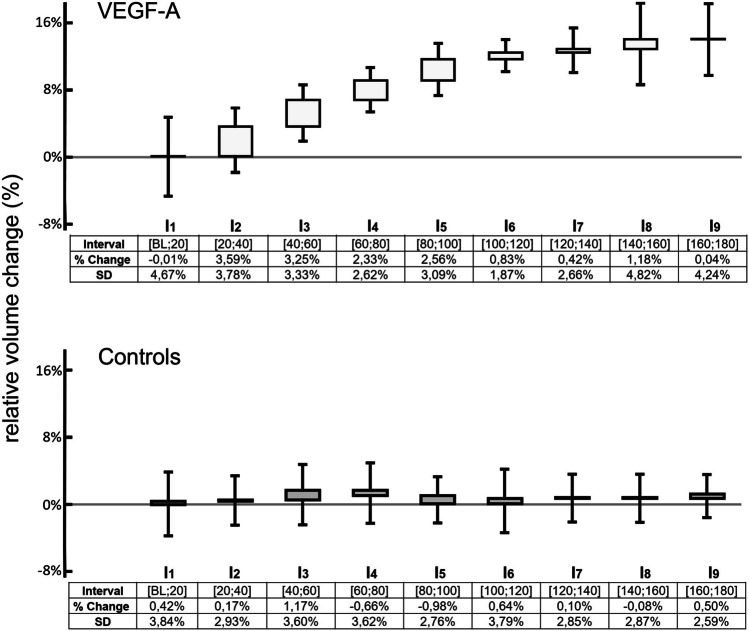
Volume increase. We compared the change in distal outflow tract volume from each time point at 20-min intervals (*n*=48). We found an early increase in volume in VEGF-A-treated eyes, especially between 20 and 100 min, before reaching a plateau at 140 min. In contrast, controls showed little change over the course of the experiment. Box, mean volume increase/decrease per interval (boxes for illustrative reasons only); SD, standard deviation

There was significant vessel dilation in eyes perfused with VEGF-A compared to controls. The mean volume increase in all VEGF-A treated eyes (*n*=24) was 16.8 ± 10.6%, whereas controls (*n*=24) showed no volume increase (0.54 ± 6.8%, VEGF-A vs. controls: p< 0.001, ANOVA, Fig. 2). The findings in AIT-treated eyes were similar to those in eyes with intact TM (VEGF-A: 17.6 ± 10.6%; VEGF-A_AIT_: 16.0 ± 11.0%; controls: −0.1 ± 6.8%; controls_AIT_: 1.2 ± 7.0%; *n*=12 each, Supplementary Data Fig. S2). Our post-hoc analysis indicated that we had a power above 99% for this volume change (data not shown). In VEGF-A-treated eyes (*n*=24), we observed a strong increase in vessel volume between 40 and 100 min (20–40 min: 3.59% ± 3.78%, 40–60 min: 3.25% ± 3.33%, 60–80 min: 2.33% ± 2.62%, 80–100 min: 2.56% ± 3.09% ) after the start of VEGF-A perfusion (Fig. 3). In contrast, there were little to no changes in the control eyes (Min: −0.98% ± 2.76%, Max: 1.17% ± 3.60%, *n*=24).

We confirmed our results by applying repeated measures (RM) ANOVA with Greenhouse-Geisser correction. The volume of the distal outflow tract differed significantly over time in eyes treated with VEGF-A (*n* = 24; F(1.961, 45.105) = 24.018, *p* < 0.001, RM ANOVA), but not in control eyes (*n* = 24; F(2.076, 43.596) = 1.084, *p* = 0.349, RM ANOVA). To analyze potential interactions between AIT and VEGF-A, we conducted a two-way ANOVA. There was no significant interaction of AIT and VEGF-A on distal outflow tract volume (F(1, 44) = 0.304, *p* = 0.584, two-way ANOVA). The analysis again confirmed a significant impact of VEGF-A on distal outflow tract volume (*p* < 0.001, two-way ANOVA), and determined that AIT itself had no effect on distal outflow tract volume (*p* = 0.967, two-way ANOVA).

### VEGF-A increases the outflow facility

**Fig. 4:**
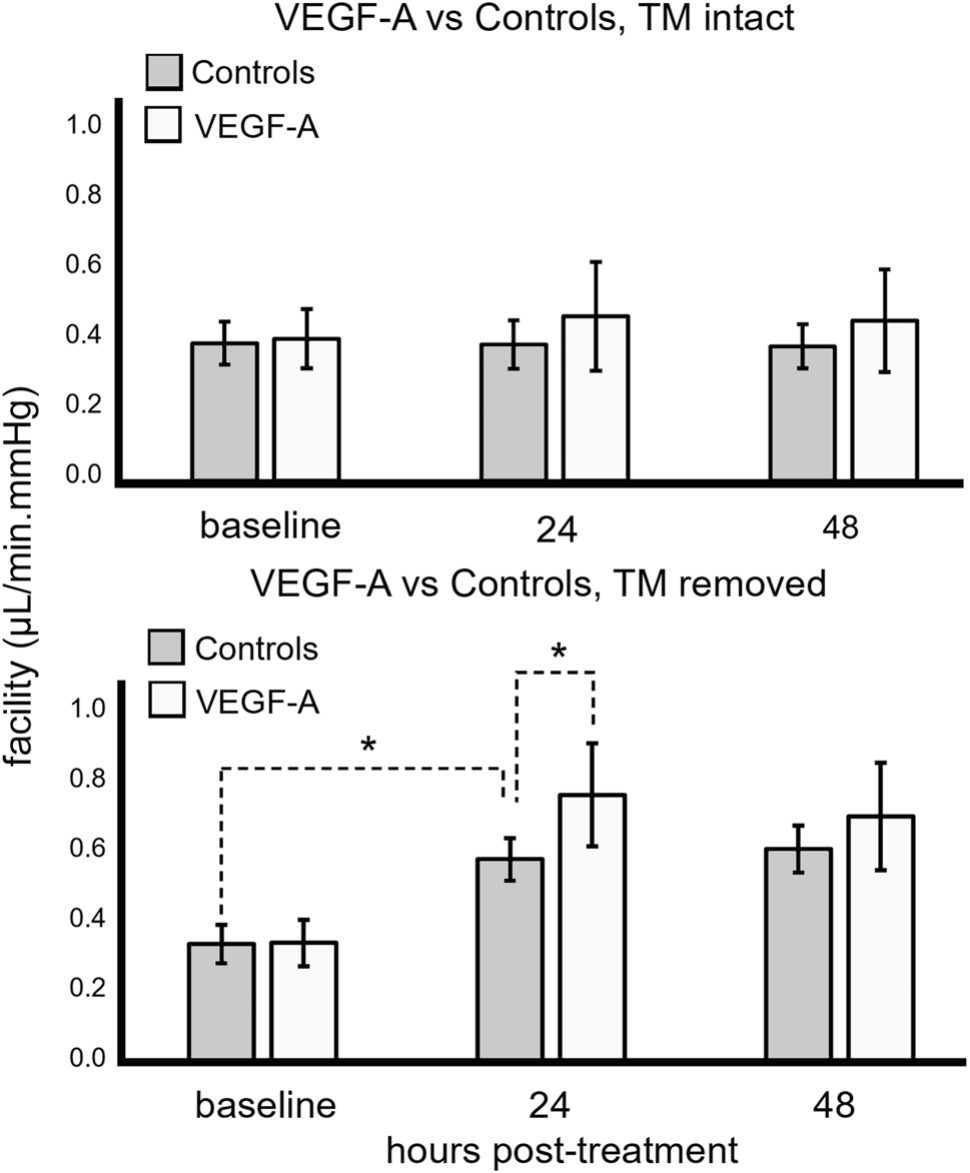
Effect of VEGF-A on outflow facility. The facility is shown for controls and VEGF-A treated eyes without (top bar) and with AIT (bottom bar) after baseline evaluation. We found a significant increase in outflow facility in eyes after AIT for the treatment and control groups. Compared to controls, VEGF-A-treated eyes showed significantly higher outflow facility 24 h after AIT. Bar graph, mean outflow facility; Whiskers, SD; *p<0.05, ANOVA

The mean baseline IOP in all groups was 18.83 ± 3.35 mmHg. There were no differences in baseline outflow facility between groups (controls 0.323 ± 0.050 μl/min×mmHg, controls_AIT_; 0.327 ± 0.069 μl/min×mmHg, VEGF-A 0.334 ± 0.054 μl/min×mmHg, VEGF-A_AIT_ 0.330 ± 0.065 μl/min×mmHg, all *p*>0.05, ANOVA). AIT significantly increased outflow facility at 24 and 48 h in VEGF-A treated eyes as well as in controls (Fig. 4).

VEGF-A significantly increased outflow facility in VEGF-A_AIT_ (0.783 ± 0.144 μl/min×mmHg) compared to controls_AIT_ (0.565 ± 0.128 μl/min×mmHg). After 24 h, this increase was 137.46% (VEGF-A_AIT_) compared to 72.92% (controls_AIT_, *p*<0.05, ANOVA). These changes were not significant after 48 h (*p*=0.085, ANOVA; VEGF-A_AIT_: 0.748 ± 0.151 μl/min×mmHg, 126.87%; controls_AIT_: 0.593 ± 0.120 μl/min×mmHg, 81.56%). In eyes with intact TM, we also observed a slightly higher outflow facility in the VEGF-A group (24 h: 0.386 ± 0.060 μl/min×mmHg, 15.82%; 48 h: 0.376 ± 0.066 μl/min×mmHg, 12.62%) compared to controls (0.320 ± 0.057 μl/min×mmHg, −1.10%; 48 h: 0.316 ± 0.052 μl/min×mmHg, −2.25%), but the differences were not statistically significant (*p*>0.05, ANOVA).

## Discussion

In this study, we tested the hypothesis that VEGF-A could dilate the distal outflow pathway and increase outflow facility. The SD-OCT and outflow facility data presented here show that this is indeed the case. Several studies have shown a surprising effect of the distal outflow tract on IOP in different species, including mouse [24], porcine [9–11], and human eyes [9]. In these, outflow resistance could be reduced with agents such as nitric oxide (NO) [9, 10] and rho-kinase inhibitors [11], which cause vasodilation. VEGF-A can also dilate blood vessels. It does so by binding to VEGF receptors 1 and 2 on the surface of vascular endothelial cells [25]. This activates the PI3K/Akt/eNOS signaling pathway to produce NO [26–28], a potent vasodilator that relaxes vascular smooth muscle cells, increasing their diameter and improving flow [29]. Fujimoto et al. found that VEGF-A can lower IOP [16], but it remained unclear by what mechanism and at what level of the conventional outflow system. Conversely, Reina-Torres et al. showed that blocking VEGF-A can increase IOP [15].

The SD-OCT data of the current study indicated that constant pressure perfusion allowed for stable morphology throughout the observation period. VEGF-induced outflow tract vasodilation was independent of the presence or absence of TM and the different flow and shear forces that these eyes may have experienced. We were concerned that AIT in control eyes might lead to eNOS release and vasodilation, but this did not occur. In contrast, VEGF-A caused an early increase in intravascular volume before plateauing. This is consistent with our previous results using NO [10], but differs somewhat from the slower vasodilation in another study in the same model where we examined netarsudil, a rho kinase inhibitor. In those experiments, the kinetics were slower, reaching a maximum at 180 min [11].

To assess whether these morphologic changes could improve the aqueous humor outflow facility, we performed continuous IOP monitoring for 72 h. Interestingly, VEGF-A caused an average increase in outflow facility after 24 and 48 h of perfusion, but the data did not reach statistical significance due to a relatively large standard deviation. It appears that VEGF, a 46 kDa homodimer of 23 kDa subunits [30], does not diffuse through the TM efficiently enough. Therefore, it cannot affect the distal outflow pathway as effectively as in eyes without TM.

Our results are consistent with other studies on VEGF-A and aqueous outflow facility. Reina-Torres et al. described increased outflow facility in mouse eyes after VEGF-A treatment [15]. They observed an increased secretion of VEGF-A from the TM and suggested that it may exert its main effect at the level of the inner wall of Schlemm’s canal, which is directly adjacent to the TM. Our results do not point to a specific anatomic location that regulates outflow but suggest that the entire distal outflow tract may do so, even without the TM and the inner wall of Schlemm’s canal.

In conclusion, our study showed that VEGF-A may act as a regulator of conventional outflow in the eye, causing vasodilation of the distal outflow tract and subsequently lower IOP. Further research is needed to identify the specific anatomic sites that most influence outflow.

### Supplementary information

ESM 1 **Fig. S1** Short clip showing the full 3D reconstructed vessel morphology at the same scale as in Fig. 1 (Link to video). (MP4 108179 kb)

ESM 2 **Fig. S2** Distal outflow tract volume changes**.** Volume changes are shown for all eyes relative to their baseline volume (n=48). Vessel volume increase was significantly higher in VEGF-A treated eyes (treatment group = T) with and without preserved TM. Controls (C) showed little or no change regardless of TM ablation. P-values were calculated to compare the VEGF-A group with the corresponding control group. Box-plot indicates the relative volume change, x=mean, SD= standard deviation; *=p<0.001, ANOVA. (PNG 157 kb)

## Data Availability

Data is available from the corresponding author on request.

## Declarations

### Research involving animals

Porcine eyes were obtained from a local abattoir. No Institutional Animal Care and Use Committee approval was required because no animals were harmed or killed for the purpose of doing research. The eyes were transported and processed in compliance with the European regulation EC No. 1069/2009 (animal by-products regulation). They were kept in a hygienic and cool environment in a disposable insulated box immersed in potassium buffered saline. Before being dissected, they were disinfected with 5% povidone-iodine ophthalmic solution.
