# Supplementary figures and images for: VEGF-A-induced changes in distal outflow tract structure and function
Source: Graefes Arch Clin Exp Ophthalmol. 2023 Oct 13;262(2):537–43. doi: 10.1007/s00417-023-06252-5 (PMC10844149; doi:10.1007/s00417-023-06252-5)

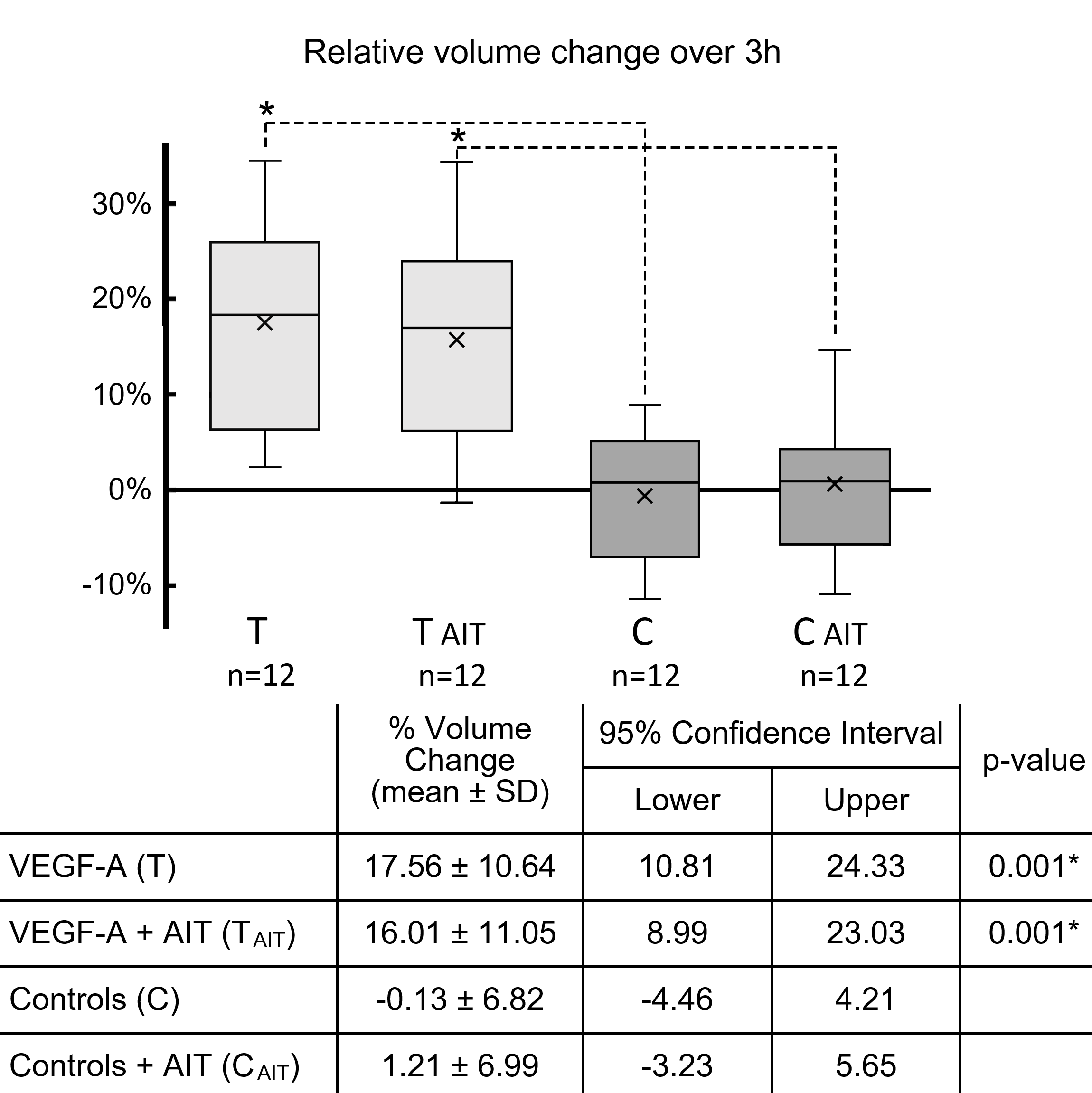

Supplement: Supplementary file 2 — Fig. S2 Distal outflow tract volume changes. Volume changes are shown for all eyes relative to their baseline volume (n=48). Vessel volume increase was significantly higher in VEGF-A treated eyes (treatment group = T) with and without preserved TM. Controls (C) showed little or no change regardless of TM ablation. P-values were calculated to compare the VEGF-A group with the corresponding control group. Box-plot indicates the relative volume change, x=mean, SD= standard deviation; *=p<0.001, ANOVA. (PNG 157 kb) [file 417_2023_6252_MOESM2_ESM.png]
